# The potential of a lecture series in changing intent and experience among health professionals to conduct research in a large hospital: a retrospective pre-post design

**DOI:** 10.1186/s12909-019-1548-4

**Published:** 2019-05-02

**Authors:** Michelle McNab, Angela Berry, Tony Skapetis

**Affiliations:** 1 Oral Health, Western Sydney Local Health District, Oral Health Network Offices, Level 2 Westmead Centre for Oral Health, Westmead, 2145 Australia; 2 University of Sydney Faculty of Medicine and Health, Sydney Dental School, Mons Road, Westmead, 2145 Australia; 3 Intensive Care Unit, Westmead Hospital, PO Box 533, Wentworthville, 2145 Australia

**Keywords:** Research capacity, Graduate education, Continuing education, Research training, Education program, Research intent, Research experience

## Abstract

**Background:**

Promoting research capacity within public health can encourage and engage employees to undertake research, utilising their understanding of the complex needs that exist within the public health system to provide more relevant research outcomes. Despite this, there are a number of reasons cited by health care professionals as to why research is not undertaken, and a lack of support for research participation results in missed opportunities for experienced clinical and public health staff to gain research experience, expand the evidence base, and promote and support research. The aim of this study is to identify if education in research, delivered through a series of lectures at a large tertiary referral hospital, results in an increase in the experience and intent to conduct research.

**Methods:**

A series of six lectures to aid in the understanding and development of research were delivered to health employees, health care professionals, students and their associates within a large public Australian hospital. Following these lectures, a validated instrument was developed and asked respondents to assess their research activity, research training history, and experience in conducting research using a retrospective pre/post- test design.

**Results:**

Over half (57.1%) of respondents (*n* = 49) reported no previous researcher education training prior to the lectures. Following the lectures, reported researcher experience increased significantly in the areas of writing a research protocol, using qualitative research methods, publishing research, writing and presenting a research report, analysing and interpreting results, using quantitative research methods, generating research ideas, and applying for research funding. At 6 months following the lecture series intent to be involved in further research was seen in the areas of submitting an ethics application, analysing qualitative and quantitative research data, and research funding applications.

**Conclusions:**

Six one hour face to face research lectures can improve self-reported levels of intention to become involved in research as well as research experience amongst hospital health care professionals at 6 months. This traditional modality of education should still be considered as relevant strategy in building research capacity as measured innovatively using a retrospective pre/post test methodology.

## Background

In the health care environment research is fundamental and participation in research education can be beneficial in up-skilling and developing traits for individuals to undertake research projects. Promoting research capacity within public health can encourage and engage employees to undertake research, utilising their background and understanding of the complex needs that exist within the public health system to provide more relevant research outcomes. Studies have also found that the development of a research program results in improved cultural attitudes to research and productivity [1].

Despite this, there are a number of reasons cited by health care professionals as to why research is not undertaken, including time constraints, lack of suitable mentors or role models, lack of development of research skills during training, lack of funding and support from management, and lack of perseverance to complete research projects [1–5]. This lack of support for research participation results in missed opportunities for experienced clinical and public health staff to gain research experience, expand the evidence base within their fields, and promote and support research for others. A change of workplace culture to promote one which is research focused requires a high level of support, both financial and managerial, encouragement, and leadership [6]. Exposure to research in the early parts of health professionals’ careers, removing financial barriers, and allowing for continued participation in research activities can instigate continued research later in their careers [2].

While a great deal of funding goes into researcher development, there is not a lot known about what educational and training techniques can alter researcher behaviour, or the assessment and evaluation of individual strategies and their outcomes [7, 8]. Mentorship, multifaceted interventions, and self-assessment activities have been indicated as possible tools for effective research training, but these options are largely time and personnel reliant and may not be desirable for those who have an early interest in research and want to gain background information [8–10].

A simple alternative that is commonly adopted in delivering researcher education material is through lecture format, which is a traditional and cost-effective approach well suited to the public health sector. Some studies suggest that face-to-face lectures offer a still relevant alternative to other forms of education delivery which should continue to be considered as a viable option [11, 12]. It is this approach that was taken at a tertiary referral hospital, to deliver a lecture series in researcher education training. The aim of this study is to identify if training in research, delivered through this series of lectures, results in an increase in experience and intent to conduct research.

## Methods

Western Sydney Local Health District (WSLHD) is one of the fastest growing areas of New South Wales (NSW), Australia, with more than 1.3 million residents estimated by 2031. WSLHD employs more than 13,000 staff across several sites, of which Westmead Hospital is the principal tertiary referral hospital. A series of six face to face lectures to aid in the understanding and development of research were delivered to health employees, health care professionals, students and their associates within Westmead Hospital. These were developed and organised by the Research and Education Network which provides support for research and training for WSLHD. The education intervention involved a single face-to-face delivery of a series of lectures 1 hour each in duration, consisting of an introduction, purpose and definition of research, conducting research, ethics and governance, as well as presentation and publication of research. Timing of the lectures was a matter of convenience and did not correspond to the commencement of new staff. The lectures were delivered fortnightly over a period of 10 weeks. The lectures were advertised through staff electronic communication systems and further disseminated to associates of employees through email.

The study had full ethics approval from the Western Sydney Local Health District Human Research Ethics Committee (HREC) LNR/16/WMEAD/457. Attendance to the lecture series was voluntary and participants who attended the lectures agreed to be contacted by email 6 months following completion of the lecture series to complete a 6 page questionnaire. The questionnaire survey instrument comprised of 4 sections each previously validated. The first consisting of demographics [13]. The second measured current research activity and professional qualifications [14] and research training history [13]. The third and fourth sections measured self-assessed experience in and intent to conduct research [10, 15] using a retrospective pre/post-test design where participants complete both pre and post questions at one time point i.e. 6 months following the intervention activity [16]. The questionnaire was piloted amongst 8 WSLHD clinicians and several minor changes were made to help validate the survey. As per the Merriam-Webster dictionary, intent was defined as “the act or fact of intending: purpose”, and experience was defined as “practical knowledge, skill, or practice derived from direct observation of or participation in events or in a particular activity” [17].

A sample of forty eight participants was required based on power calculations to achieve a statistical power of 90% similar to other studies [18, 14, 15].

Data was analysed using SPSS version 25.0 for descriptive analysis and examined for frequency distribution. Cross tabulations were performed using McNemar’s Test to identify associations between groups of data collected, and a value of *p* < 0.05 was considered statistically significant. Data was de-identified and the Wilcoxon Signed Ranks Test used on matched pair responses from pre/post test self-assessment questions.

## Results

The total number who attended one or more lectures in the series in person was 160 people. Of these 126 consented to be contacted after 6 months, with 49 participants completing the survey. The response rate for the study was 38.9%.

Demographic results are presented in Table 1 with the majority of participants female (89.8%) and over two thirds (67.3%) holding postgraduate qualifications (Table 1).

**Table 1:** Participant Demographics and Research and Education History

*Variable*	*n*	*Percentage %*
Age		
< 35 years	12	25.0
35–49 years	13	27.1
50–65 years	23	47.9
Gender		
Male	5	10.2
Female	44	89.8
Professional Qualifications		
Certificate	1	2.0
Undergraduate	15	30.6
Postgraduate	33	67.3
Years Since Graduation		
N/A	2	4.1
< 5 years	9	18.4
5–10 years	9	18.4
11–15 years	6	12.2
> 15 years	23	46.9
Previous Researcher Training or Education		
Yes	21	42.9
No	28	57.1

As demonstrated in Table 2, the participants came from a wide range of backgrounds with the largest groups reporting a background in Allied Health (43.8%), followed by Nursing (27.1%).

**Table 2:** Disciplines of Participants

*Discipline*	*n*	*Percentage %*
Administration	2	4.17
Allied Health	21	43.75
Epidemiology	1	2.08
Health Manager	2	4.17
Hospital Scientist	1	2.08
Library Information	1	2.08
Manager	2	4.17
Medicine	1	2.08
Multicultural Health	1	2.08
Nursing	13	27.08
Paediatrics	1	2.08
Research	1	2.08
Women’s and Newborn Health	1	2.08

**Fig. 1:**
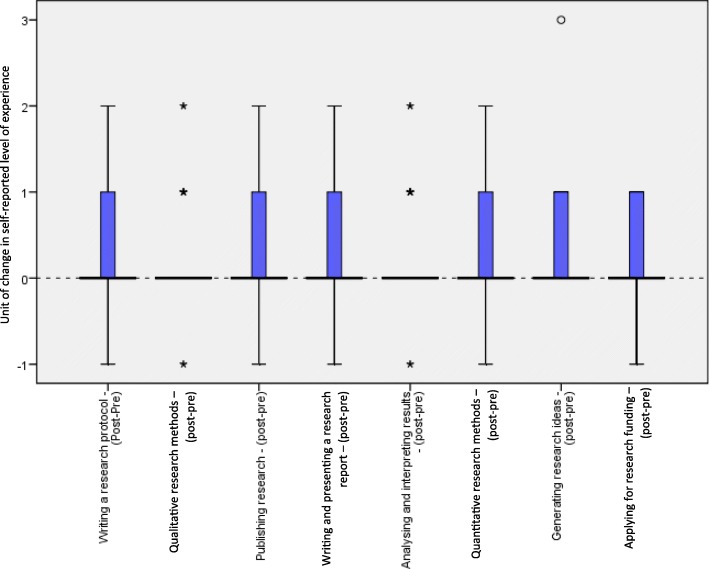
Change in level of experience across different areas of research

Over half (57.1%) of respondents reported no previous researcher education training prior to the lectures. Of those that had previous researcher education training, the majority (32.7%) had received it in the form of lectures. Using the retrospective pre-test to assess experience prior to attending the lecture series, more than half of participants (60.0%) reported having no experience in writing a research protocol, while at post-test this number had reduced to 39.1% (*p* < 0.01). A similar reduction in lack of experience was seen in those reporting no experience in the retrospective pre versus post-test for using qualitative research methods (34.8 to 25.6%, *p* < 0.05), publishing research (65.2 to 50.0%, p < 0.05), writing and presenting a research report (50.0 to 38.6%, *p* < 0.03), analysing and interpreting results (34.8 to 23.3%, *p* < 0.03), using quantitative research methods (41.3 to 27.3%, *p* < 0.01), generating research ideas (24.4 to 10.9%, *p* < 0.01), and applying for research funding (65.2 to 51.1%, *p* < 0.04) as seen in Fig. 1.

**Fig. 2:**
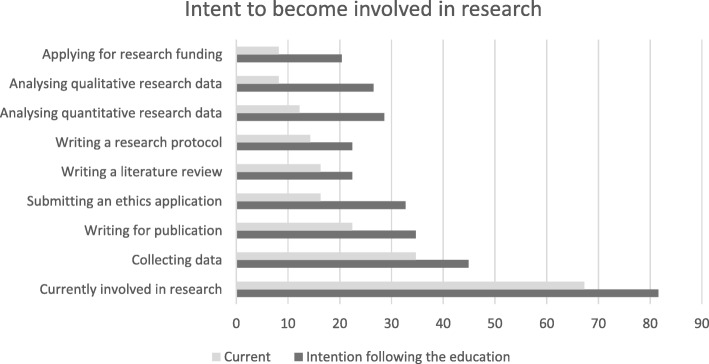
Current involvement in research and intent to become involved in research

Results concerning intent to be involved with research beyond the 6 month survey point (Fig. 2), showed significance in the areas of submitting an ethics application (20%, *p* < 0.02), analysing qualitative research data (21%, *p* < 0.01), analysing quantitative research data (20%, *p* < 0.02), and applying for research funding (14%, *p* < 0.03).

## Discussion

This study assessed changes in experience and intent to conduct research following a training intervention, delivered through a series of traditional lectures. Other forms of lecture education delivery exist, such as through video, active learning, online accessible content, and online module delivery which, dependent on context, may be more effective than traditional face-to-face lectures. The latter may still be relevant as they provide an opportunity for real-time questions and networking with both peers and educators which is essential in a multidisciplinary health environment. In addition to this, in comparison to mentorship and full curriculum development, it is a cost effective approach. Responses showed that more than two thirds (67.3%) of participants were currently involved in research 6 months after completion of the education intervention. There was significance recorded in the intent to become involved in several aspects of further research, indicating an ongoing interest and involvement in research activities following the intervention.

The 6 month interval between the time of lecture delivery and survey was specifically chosen to allow for the development of research activities by participants, which is consistent with other similar published studies [18] although it should be noted that confounders such as participant recall could have affected results. Significant results included intent to become involved in the research areas of ethics application, analysis of both qualitative and quantitative research data, and application for research funding. This represents intent for involvement in a wide range of early research phases.

As research stages can take lengthy periods of time, involvement in later stages of research, such as writing reports or papers for publication, or writing a literature review, may have been limited by the 6 month follow-up. Further involvement in these later stages may be evident if a later follow-up was conducted and this may be a consideration for future studies.

The majority of participants were female (89.8%), which could reflect the staffing patterns of public hospitals, including the workforce in this local government health district which reports 73% female workforce representation [19]. In general, there is a higher representation of women in health care, including the professions of Nursing and Allied Health [20], which may have resulted in some element of gender bias. Although this is representative of the Public Sector workforce in NSW Health which has 74.6% female representation [21]. A limitation of this study is that this heavily female participant cohort may not be reflective of other health districts or in other countries, and this may affect study results in replication of the study in areas where male to female proportions are much greater.

Participants were also older, with almost half (47.9%) above the age of 50 years. It has been reported that previous degrees were less likely to include research methods in their curricula hence the greater interest among older clinicians [22]. Two-thirds of participants had completed postgraduate qualifications, and almost half (46.9%) had graduated more than 15 years ago. Several of these qualifications were reported to include a research component, although fewer than half (42.9%) had undertaken previous researcher training or education. This could indicate that either a proportion of their postgraduate qualifications were not research based, or they did not consider experience in research during these studies as researcher training. In fact, other training including postgraduate education accounted for 12.2% of previous researcher training. The highest reported previous training was received through lectures (32.7%), indicating the popularity of lecture based researcher training.

Significant change in the self-assessed level of experience was seen in a wide range of research areas, indicating the effectiveness of the lecture series in improving researcher experience. The increase seen in writing a research protocol was significant, as this is one of the first stages of research that is undertaken. Prior to the education intervention, more than half of participants (60.0%) reported having no experience in writing a research protocol, which reduced considerably to 39.1% following the lectures. This indicates that the lecture series was effective in educating attendees on the steps required in the core stage of planning and commencing a research project.

The lecture series was not compulsory for any staff, therefore it is likely those who attended had a previous interest in research, which may account for some of their involvement and intent to conduct further research. However, the lectures may have acted as a catalyst in fostering that intent, and providing the knowledge and education to initiate a research project.

The innovative retrospective pre/post-test methodology was used for this study due to its effectiveness, efficiency and ease of administration. Participants rate themselves with a single frame of reference on both pre-test and retrospective post-test which helps to reduce response shift bias [16]. It was also utilised in a busy hospital setting due to its advantage of a single administration and implementation of the study design post-intervention [16]. In the analysis of the data a paired analysis was chosen for its effective simplicity and could be considered a limitation compared to other more complex analysis, such as multivariate modelling.

The response rate for the study was relatively low, at 38.9%. Although this was to be expected as it sits within the range of 33 to 56% as seen in previous Australian surveys [23, 24]. Several steps were taken to try to improve the response rate, including assurance of anonymity, use of a brief and easily accessible survey tool, explanation of the impact of their contribution to the research, and several contact reminder attempts [24]. While further improvement in response rates may have been gained through incentives, telephone or face to face follow up interviews [25], these were not practical options for this study. The choice for a pre/post-test design was made over other, potentially more preferable, experimental designs due to two main factors. The first was the development of the study following the completion of the lecture series, thus removing the possibility of a pre-test prior to the intervention. The second is due to the small number in the study sample which did not allow inclusion of a control group while still maintaining statistical power. Future studies in this area may consider the use of other experimental designs.

While the results of this study do not provide ground breaking advancements, within the context of the current climate of education delivery where there is growing pressure to move away from the face-to-face lecture format, this study demonstrated and helps reinforce that this style of lecture series is still relevant in a modern health education environment.

A further limitation of this study is that the lecture series based format of education delivery was not directly compared to other delivery methods, nor the questions of how and why of success investigated, as these may be useful to consider in future studies.

It might also be useful to conduct further follow up in future studies to identify the long-term effects of the education intervention on the participants’ experience in and intent to conduct research, in particular those aspects of research that take longer time periods to complete, such as grant applications and writing for publication. This could provide a better insight into the effect of the intervention on these areas of research. We also recommend in future research concerning this lecture series and its participants that the inclusion of the type of research that participants engaged in following the intervention should be considered. Further research in delivering the intervention with accompanying support, such as workshops, tutorials, or mentorship, might also be useful in assessing whether such modalities improves experience in and intent to conduct research when compared to lectures alone.

## Conclusion

Six one hour face to face research lectures resulted in an improvement in self-reported levels of intention to become involved in research as well as research experience amongst a group of health care professionals within a large tertiary referral hospital at 6 months. This traditional yet still relevant modality of education should still be considered as an effective strategy at building research capacity when measured innovatively using a retrospective pre/post-test methodology.

## Abbreviations

NSW: New South Wales
WSLHD: Western Sydney Local Health District
